# Social engagement, pleasure, and memory in musical reminiscence workshops for individuals with Alzheimer’s disease

**DOI:** 10.3389/fnhum.2026.1803210

**Published:** 2026-04-22

**Authors:** Mikael Genguelou, Hervé Platel, Cécile Bourgeois, Marie Gambonnet, Sophie Bayard

**Affiliations:** 1 Univ Paul Valéry Montpellier 3, EPSYLON EA 4556, F34000, Montpellier, France; 2 Inserm, U1077, EPHE, Université de Caen Normandie, Normandie Université, PSL Université Paris, CHU de Caen, GIP Cyceron, Neuropsychologie et Imagerie de la Mémoire Humaine (NIMH), Caen, France

**Keywords:** Alzheimer, autobiographical memory, emotions, music, music therapy, pleasure, social interaction

## Abstract

**Introduction:**

Social bonding is essential in Alzheimer’s Disease (AD), as social withdrawal reduces quality of life and can worsen Behavioral and Psychological Symptoms in Dementia. Music therapy offer a promising approach. This study examines the effects on social engagement in AD and explores links between verbal interaction, memory, and emotion.

**Methods:**

Nineteen voluntary residents with moderate to severe AD from four nursing homes participated. Nine musical reminiscence workshops were conducted. A single-group intervention study was conducted, with assessments at baseline, three points during the intervention, post-intervention, and one-month follow-up. Emotions were assessed using the Observed Emotion Rating Scale. Social engagement was measured via the social interaction domain of the Alzheimer’s Disease-Related Quality of Life scale. Episodic memory was evaluated with the simplified Tempau Test and an observational grid. Reminiscences and verbal interactions during workshops were counted.

**Results:**

Verbal interactions and memory episodicity increased across workshops. Pleasure correlated positively with interaction frequency. Daily social engagement also improved after the intervention.

**Discussion:**

Musical reminiscence workshops enhance autobiographical memory and foster social engagement. Pleasure appears to be associated with social interactions. Music interventions can improve the social quality of life in people living with Alzheimer’s disease.

## 1 Introduction

Throughout the course of Alzheimer’s Disease (AD), alongside cognitive difficulties, the impact on emotional and social life is significant, with frequent occurrences of apathy, emotional blunting, and social withdrawal (Leone et al., 2012; Porcelli et al., 2019). This lack of interaction is problematic because social engagement is a basic need for human well-being, including that of older people (Bruggencate et al., 2018). Social engagement appears to be protective, evidence indicates that a high social engagement among institutionalized individuals with neurocognitive disorders is negatively associated with reduced autonomy, poorer cognitive and visual functioning, and, most notably, to higher levels of depression (Kang, 2012). As the disease progresses, social needs become increasingly unmet, especially in highly dependent individuals, which may contribute to Behavioral and Psychological Symptoms in Dementia (BPSD). Indeed, Cohen-Mansfield et al. (2015), reported a link between loneliness and verbal agitation in dementia. These findings underline the necessity of strategies that actively foster social engagement in people with AD.

Music therapy is defined by the World Federation of Music Therapy (WFMT) as “the professional use of music and its elements as an intervention in medical, educational, and everyday environments with individuals, groups, families, or communities who seek to optimise their quality of life and improve their physical, social, communicative, emotional, intellectual, and spiritual health and wellbeing.” [World Federation of Music Therapy (WFMT) (2026)]. Another important aspect is that interventions in music therapy are generally individualized (Bradt et al., 2021). In people with dementia, music therapy has been shown to provide multiple benefits, ranging from the maintenance of cognitive function to the reduction of BPSD (Genguelou et al., 2026).

A unique feature of listening to music is its well-known ability to foster reminiscence, even in individuals with AD, where memory impairment is the primary symptom (Belfi and Jakubowski, 2021; El Haj et al., 2012; Irish et al., 2006). Importantly, although memory retrieval declines early in Alzheimer’s disease, musical memory often remains relatively preserved, even in later stages (Jacobsen et al., 2015).

At early stages, music listening facilitates autobiographical recall more effectively than explicit retrieval in silence (El Haj et al., 2012), and improves verbal narration (El Haj et al., 2013), particularly when the music is familiar and emotionally salient (Cuddy et al., 2015). Involuntary memories, often vivid, specific, and positive, may play a central role in this effect (Cuddy et al., 2017). To improve the quality of life of individuals with AD, enhancing autobiographical memory is particularly relevant because of its strong connection to personhood (Petherbridge, 2025). Self-defining memories (SDM) are highly significant autobiographical memories that play a central role in an individual’s life story, identity, and sense of self-continuity even in AD at a mild stage (El Haj and Allain, 2020). In this context, the ability of music to elicit autobiographical reminiscence may represent a particularly valuable avenue for promoting psychosocial well-being in individuals with AD. By facilitating access to SDM and supporting the continuity of identity, music-based interventions may help sustain social engagement. Consistent with this perspective, Platel et al. (2021) reported that the sense of identity remained stable during musical reminiscence workshops even in the moderate to severe stages of AD (Platel et al., 2021). In the study, involving 20 residents with moderate to severe AD in nursing homes they found that participation in a six-session program of musical reminiscence workshops led not only to an increase in the number of autobiographical memories recalled but also to improvements in their episodic quality, with participants providing more detailed recollections by the end of the intervention. They further reported a significant increase in verbal interactions among participants and the workshops were characterized by frequent smiling and humorous exchanges. Although the intervention did not enhance the sense of identity, it was associated with increased social interactions.

In this context, musical reminiscence therapy appears particularly promising, as it supports both autobiographical memory and social engagement during the reminiscence workshop (Kelly and Ahessy, 2021). In a study involving residents with dementia, Ashida (2000) reported qualitative observations of enhanced interaction skills during and immediately following group reminiscence music therapy sessions. After musical reminiscence workshops with people with dementia, Evans et al. (2019) observed mixed outcomes: some participants showed improved social interaction in the sessions and in daily life, while others exhibited social engagement improvements only during the workshop sessions. Del Mastro et al. (2021) propose that episodic retrieval during musical reminiscence is a key mechanism. Autobiographical memories foster opportunities for social sharing through peer exchanges and intergenerational dialogue. To our knowledge, no empirical study has yet confirmed this theory.

Besides the memory-related effects, another explanation for the social benefits of music may lie in the pleasure it induces. Music’s ability to evoke emotions can trigger pleasure responses of varying intensity. Listening to music engages the reward system, leading to dopamine and endogenous opioid release, which in turn promote relaxation and analgesia (Blood and Zatorre, 2001). These neurobiological mechanisms may contribute to social bonding during shared music experiences (Tarr et al., 2014). Although evidence in AD remains limited, it is plausible that pleasure derived from music enhances mood and, in turn, facilitates social engagement.

Building on these findings and the recognized need to foster social engagement in Alzheimer’s disease, the present study investigates the potential contribution of musical reminiscence interventions to social functioning. Autobiographical musical memories and musical enjoyment have been identified as two mechanisms closely linked to the social dimension of music (Nummenmaa et al., 2021). Accordingly, particular attention is given to the potential role of autobiographical memory retrieval and the experience of pleasure in fostering social interaction during musical reminiscence workshops.

Although music-based interventions have been shown to enhance social engagement among individuals with neurodegenerative diseases and their caregivers, the underlying mechanisms linking memory processes, emotional responses, and prosocial behaviors remain insufficiently understood (Platel et al., 2021; Colverson et al., 2022). Given the established links between episodic memory and social cognition (Klein et al., 2009), an important question is whether the retrieval of musical autobiographical memories may support social interaction and help mitigate social isolation, even at advanced stages of Alzheimer’s disease. In addition, it remains unclear whether the social engagement observed during such interventions extends beyond the workshop context and relates to participants’ social interactions in everyday life.

To address these issues, the present study examines the effects of repeated musical reminiscence workshops on the social engagement of nursing home residents with moderate to severe Alzheimer’s disease. Social engagement is assessed through the analysis of verbal interactions among participants during group sessions. We further investigate whether social interaction is associated with two key factors: (1) the quantity and quality of autobiographical memories retrieved, and (2) the level of pleasure experienced during the sessions. Finally, we explore whether participation in these workshops is related to social interactions in participants’ daily lives. Based on this framework and on previous literature, we formulated several hypotheses regarding the relationships between autobiographical memory retrieval, musical pleasure, and social interaction.

### 1.1 Hypotheses

First, social engagement within the workshops was expected to increase over time, reflected in a progressive rise in verbal interactions across reminiscence sessions. Similarly, increases were expected in both the number of autobiographical memories evoked and the quality of the memories recalled across sessions. The level of expressed pleasure was also expected to progressively increase during the workshops.

Second, social interaction would be positively associated with both memory retrieval and emotional experience during the sessions. Specifically, positive correlations were hypothesized between the number of verbal interactions and (a) the number of recalled memories, (b) the episodicity of retrieved memories, and (c) the level of expressed pleasure.

Third, autobiographical memory retrieval during individual interviews was expected to improve after the workshops. Songs used during the sessions were expected to serve as effective retrieval cues, eliciting a greater number of memories and greater episodic richness than songs not presented during the workshops.

Finally, the study examined whether the social benefits observed during the workshops extended beyond the intervention context, as reflected in improvements in participants’ daily social interactions following the reminiscence workshops.

## 2 Material and methods

### 2.1 Participants

A group of 19 patients diagnosed with probable AD according to the National Institute of Neurological Disorders and Stroke - Alzheimer’s Disease Related Disorders Association (NINCDS–ADRDA) criteria were included (McKhann et al., 1984). Participants were recruited through referrals from nursing facility staff. They were volunteer residents of four different nursing homes, aged 75–100 years. See Table 1 for demographic and clinical characteristics. We assessed cognitive functioning with the Mini-Mental Status Examination (MMSE) (Ouvrard et al., 2019), and the Five-Word Test, a brief neuropsychological test assessing episodic memory in just a few minutes (Mormont et al., 2012). Residents showed major cognitive impairment (MMSE scores: 7–16, mean = 12.2). Neuropsychiatric symptoms and psychopathology were assessed using the Neuropsychiatric Inventory (Sisco et al., 2000), and music engagement with a French version of the Music Engagement Questionnaire (MusEQ) (Genguelou et al., 2026), completed independently by a family member and a professional. The MusEQ questionnaire assesses engagement in music-related activities, including listening to music and participating in musical practice.

**Table 1:** Demographic and clinical characteristics of the total sample (*N* = 19).

Total sample *N* = 19 mean ± (SD) [min − max]	
Participant characteristics	Value
Demographic variables	
Sex (Female)	79%
Age	88.60 *± 7.36 [75–100]*
Education (years)	9.63 *± 4.89 [0–17]*
Cognitive and neuropsychiatric assessment	
Mini mental status examination	12.20 *± 3.38 [7–16]*
Dubois’ five words testing	3.42 *± 3.06 [0–9]*
Neuropsychiatric inventory	23.30 *± 28.30 [0–110]*
Music engagement	
Music engagement questionnaire family	60.00 *±* 13.40 *[42–89]*
Music engagement questionnaire caregiver	*55.70 ± 15.00 [34–81]*

### 2.2 Design of the experiment

The experiment was conducted in 2024 across all institutions. The following is a description of the four phases completed over 2 months.

Phase 1: individual interview assessing autobiographical memory, with and without music, using nine musical excerpts, and evaluating social engagement in daily life.

Phase 2: beginning the week after Phase 1, participation in nine Reminiscence Workshop sessions across 3 weeks.

Phase 3: individual post-workshop assessment of autobiographical memory with and without music, plus reevaluation of social engagement, 1 to 2 days after the last session.

**Figure 1:**
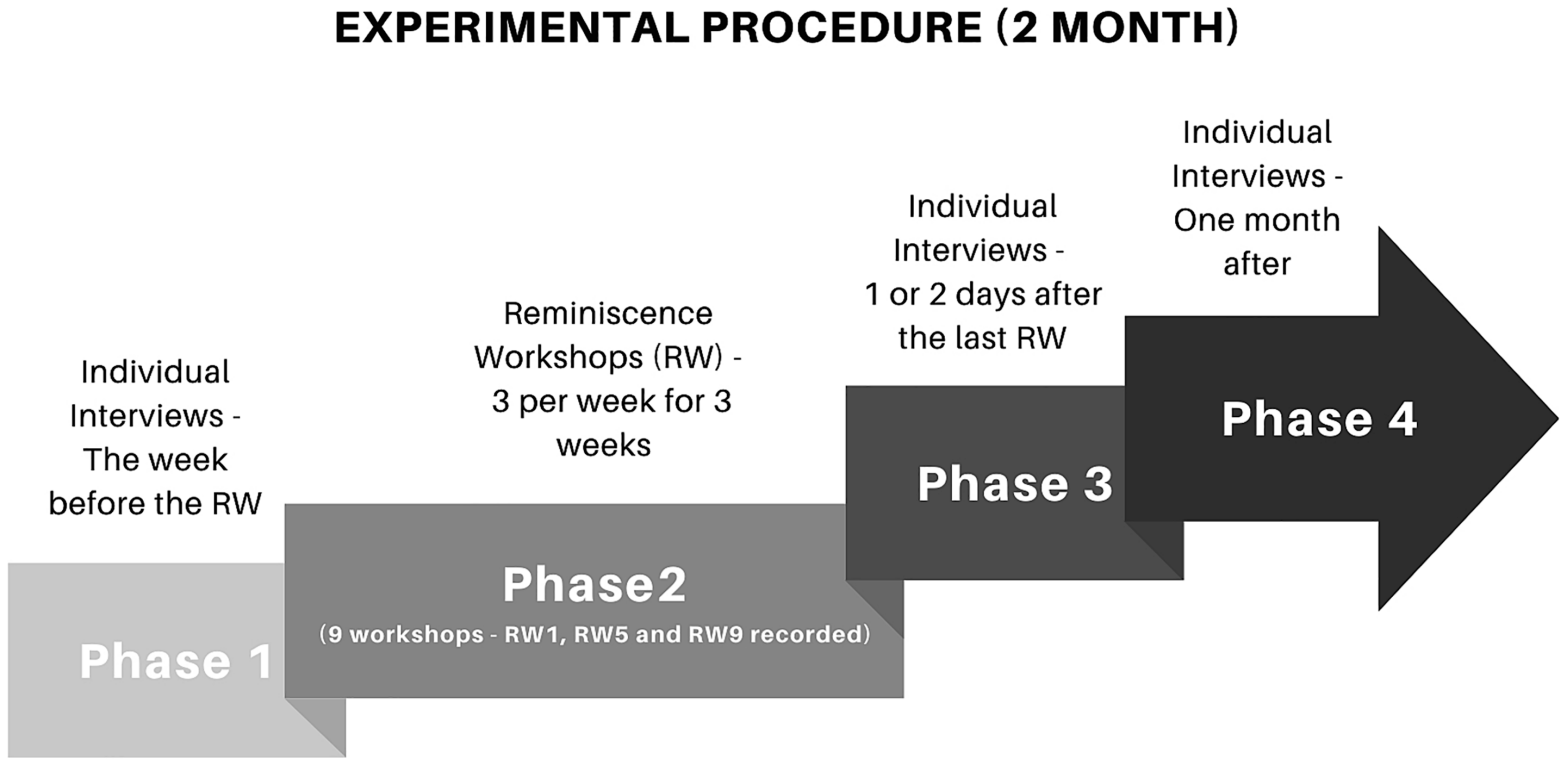
Experimental procedure of the experiment.

Phase 4: final individual memory assessment 1 month later (see Figure 1).

### 2.3 Individual interviews

At each interview, the certified psychologist (M.G.) administered the simplified TEMPau test (see hereafter). All participants completed individual interviews and post-workshop assessment. In phase 1, the participant listened to nine musical excerpts, each lasting 1 min 30 s, covering the themes of school and childhood, ballroom dancing, and love (see Table 2). These themes were selected because they are common across generations and are often associated with well-preserved memories. The study design did not enable individualized song selection; nevertheless, a pre-test was conducted to ensure that the songs were familiar and appreciated by the participants. These songs were chosen because they were popular during the patients’ youth, corresponding to the reminiscence peak observed in AD (Platel et al., 2021). During interviews, after each music excerpt, participants were asked whether the song evoked a memory. If not, up to three thematic prompts could be used (see Supplementary material). Finally, they were invited to add any further event details by asking, “Do you have any more details to share about this event?” During initial interviews with four participants, familiarity and appreciation for the songs were assessed to ensure their selection was both well-known and well-liked. Six songs were ultimately chosen for the workshops, with the three remaining songs being comparable in familiarity and appreciation.

**Table 2:** Songs used in the study, including the performer, release date, and thematic category.

Name of the song	Performer	Release date	Thematic
Douce France	Charles Trénet	1943	School and childhood
Sacré Charlemagne	France Gall	1964	
L’école est finie*	Sheila	1963	
La Java Bleu*	Fréhel	1938	Ballroom dancing
Le petit bal perdu	Bourvil	1961	
Mon amant de Saint Jean	Lucienne Delyle	1942	
Mon manège à moi	Edith Piaf	1958	Love
Marinella	Tino Rossi	1936	
Etoile des neiges*	Line Renaud	1949	

^*^Song that was not used during the reminiscence workshops.

In phases 3 and 4, the three songs most frequently played during the workshops and three other songs not used in sessions were presented during the interview (see Table 2). This enabled assessment of repeated exposure effects as a primer for memory recall.

### 2.4 Reminiscence workshop

The Reminiscence Workshops (RW) were led by a certified music therapist or two different music therapists in training. We avoided labeling the intervention as “music therapy” due to the absence of personalized song selection. Six different groups were formed, each consisting of two to four participants. Group composition was determined according to residents’ availability, and whether they could participate in the morning or afternoon sessions. The sessions were held in the same room at the same time for each group. In each session, after a welcoming period, three songs were played, each corresponding to a specific theme: school and childhood, ballroom and dancing, and love. For each theme, a participant was asked to choose between two songs. Across all sessions, each participant made an equal number of choices. After listening, each participant was asked whether the song evoked a memory. If no memory was recalled spontaneously, the music therapist asked thematic questions to facilitate recollection, such as “What games did you play at recess?” for the school period. If the memory was not situated in time or place, additional questions were asked, such as “Does it remind you of a special day?” After a participant finished recounting a memory or answering a question, another participant was invited to respond, ensuring that everyone had the opportunity to express themselves. In the event that a participant experienced difficulty recalling a memory or reported negative emotions, the music therapist provided reassurance and assessed the participant’s level of anxiety before resuming the session. Overall, no clinically significant anxiety or marked distress was observed.

#### 2.4.1 Measures during individual interviews

##### 2.4.1.1 Procedure for data collection

The interviews were fully audio-recorded for the purpose of analysis. The recordings were randomized. Two certified psychologists, who were blind to the study’s hypotheses, each scored 50% of the extracts. Initially, both psychologists scored 20% of all extracts to calculate inter-rater agreement. A collective training session was organized to minimize scoring bias. A scoring guide was produced and several coordination meetings were held.

##### 2.4.1.2 TEMPau test

A simplified version of the Episodic Autobiographical Memory Test (TEMPau test) was used to assess episodic memory across three life periods: for childhood and adolescence, participants recalled a school-related event; for young adulthood, they described an event involving someone they met during that time; and for after age 30, they recalled a family-related event (Piolino et al., 2000).

Participants were asked to recall detailed memories of specific personal events, describing when and where they occurred. They were encouraged to give as many details as possible. If spontaneous recall was absent, up to three questions were used to prompt more specific responses. Scores ranged from 0 (no or general answer) to 4 (specific event with detailed spatiotemporal context), evaluating episodic memory characteristics such as context, details (such as the particularity of the situation, thoughts, and emotions), and specificity (whether it is a single or repeated event).

##### 2.4.1.3 Reminiscences after music listening

For a memory to be included, it had to refer to a personal event, using pronouns like “I” or “we.” Non-event statements like, “I liked the music when I was a child,” were excluded. The episodicity was rated on a 12-point scale inspired by the TEMPau test (Platel et al., 2021), based on eight criteria: event uniqueness, duration under 24 h, and general and precise temporal and spatial localization (1 point each). In addition, two items (richness of contextual details and presence of emotional details) were rated on a three-point scale: one point for one detail, two points for two details, and three points for three or more details. For analysis, the highest-scored memory produced during the interview for each participant was selected. For the scoring of evoked memories during the interview, we obtained an inter-rater agreement of 79%.

#### 2.4.2 Measures during reminiscence workshops

##### 2.4.2.1 Procedure for data collection

The first, fifth, and ninth reminiscence workshops were fully video recorded to enable remote evaluation of each participant. For each participant, we analyzed two sequences of 6 min from the RW, divided as follows: Part A: The 3 min following the questions asked by the music therapist after listening to the first song on the school theme. Part B: The 3 min following the questions asked by the music therapist after listening to the second song on the dancing theme. Parts A and B were combined to create a six-minute extract for analysis. Part C: The final 6 min of each RW, corresponding to the time when participants were asked how the session was for them. This part was chosen because it provided more opportunities for spontaneous and verbal exchanges between participants. Two psychologists who were blind to the study’s hypotheses conducted the assessments by watching the video recording. The extracts were randomized, and the scoring work was divided between the two psychologists. Initially, both psychologists scored 40% of all extracts to calculate inter-rater agreement. The judges received a comprehensive scoring guide, participated in collective training sessions on scoring, and attended several coordination meetings.

##### 2.4.2.2 Verbal interactions

Four categories of verbal interaction were identified: responding to questions from the music therapist, responding to questions from other participants, spontaneous sharing relevant to the activity, and spontaneous sharing unrelated to the activity. To characterize social engagement in our analysis, we chose to sum the number of responses to other participants and instances of spontaneous sharing related to the activity. Indeed, these two types of verbal interaction reflect a meaningful engagement with the other participants in the group. We achieved an inter-rater agreement of 72%, which is comparable to the results reported in another study (Bourgeois et al., 2025).

##### 2.4.2.3 Affects measurement

We used the Observed Emotion Rating Scale to assess pleasure, anger, anxiety/fear, sadness, and interest (Lawton et al., 1999). This five-point observational scale (1 = never to 5 = more than 3 min) is valid and reliable for evaluating affect in people with dementia in nursing homes (Bourgeois et al., 2025). Negative affects (anger, anxiety/fear, sadness) were averaged into a composite score. Due to the specific nature of the RW, we chose to observe singing behavior independently when assessing the pleasure (see Supplementary materials). Our evaluation focused on the time allocated to verbal interactions during the workshop. Since music listening (which frequently promotes singing) may have been more prevalent in the first session recording, it could introduce a bias into our assessment. In the analysis, we used the expressed pleasure score excluding singing behavior. An inter-rater agreement of 93% was obtained.

##### 2.4.2.4 Reminiscences evaluation

We used the same method of data collection for evoked memories as in the individual music listening interviews. Additionally, we counted the total number of evoked memories produced during the three recorded parts, A, B and C, of each workshop. Given the severe autobiographical memory impairment observed in our sample, we retained for each participant the memory with the highest level of episodicity. This score was then averaged across participants to examine potential changes in the production of autobiographical memories with richer episodic detail. This approach was based on previous findings showing that individuals with Alzheimer’s disease tend to repeatedly retrieve the same memories while progressively enriching their episodic content (Platel et al., 2021). We obtained an inter-rater agreement of 76% for the scoring of memories during the workshops.

#### 2.4.3 Other measures

##### 2.4.3.1 Interaction in daily life

The social interaction domain of the Alzheimer’s Disease-Related Quality of Life (ADRQL), which consists of 12 statements regarding daily social relationships, was administered (Thomas et al., 2006). A professional caregiver completed the questionnaire before and after phase 2 (the workshops) (see Table 3).

**Table 3:** Experimental procedure of the experiment.

Phase 1	Phase 2	Phase 3	Phase 4
TEMPau test	Verbal interactions	TEMPau test	TEMPau test
Reminiscences after music listening	Reminiscences evaluation	Reminiscences after music listening	Reminiscences after music listening
Interaction in daily life	Affects measurement	Interaction in daily life	

### 2.5 Analyses

The majority of variables did not follow a normal distribution, absolute values of skewness greater than 3 and kurtosis greater than 10 were considered extreme (Leech et al., 2004). Consequently, all statistical analyses were conducted using non-parametric tests. The Friedman ANOVA was used to assess the progression of the variables measured across the reminiscence workshop sessions. The same analysis was applied to evaluate changes across the individual interviews.

Spearman’s correlation was employed to examine the associations between key variables across all workshop sessions. A correlation between 0.10 and 0.30 represents a small effect, a correlation between 0.30 and 0.50 a medium effect, and above 0.50 a large effect (Cohen, 1988).

The Wilcoxon test was used to compare episodicity scores from individual interviews of the Phase 3 after listening to the music excerpts used in the workshops versus those not listened to during the RW. This test was also applied to compare mean scores of social interactions before and after Phase 2 (the workshop sessions). All statistical results were considered significant at *p* < 0.05.

## 3 Results

### 3.1 Reminiscence workshops

#### 3.1.1 Verbal interactions across the RW

The Friedman ANOVA revealed an effect of the RW on verbal interactions (χ^2^ = 6.81, *p* = 0.033). Contrast analyses indicated that the number of verbal interactions was significantly higher in the 9th Workshop (mean = 25.3, min = 10, max = 51, SD = 12.1) and the 5th Workshop (mean = 22.8, min = 9, max = 38, SD = 7.48) compared to the 1st Workshop (mean = 18.5, min = 6, max = 40, SD = 8.45) with, respectively, (*p* = 0.012 and *p* = 0.041). There was no significant difference between the 5th and the 9th Workshop (see Table 4).

**Table 4:** Main results from reminiscence workshops and individual interviews.

Variable	Workshop/phase	Mean ± SD (min–max)	Contrast/comparison	*p*-value
Verbal interactions	*ANOVA (global effect)*	–	Friedman *χ*^2^ = 6.81	0.033*
	1st Workshop	18.5 ± 8.45 (6–40)	–	–
	5th Workshop	22.8 ± 7.48 (9–38)	vs 1st	0.041*
	9th Workshop	25.3 ± 12.1 (10–51)	vs 1st	0.012*
Number of evoked memories	*ANOVA (global effect)*	–	Friedman *χ*^2^ = 3.31	0.191
Episodicity of evoked memories	*ANOVA (global effect)*	–	Friedman *χ*^2^ = 6.89	0.032*
	1st workshop	4.11 ± 3.35 (0–9)	–	–
	5th workshop	4.06 ± 3.42 (0–11)	–	–
	9th workshop	5.68 ± 2.83 (0–10)	vs 1st and 5th	0.017/0.022*
Expressed pleasure	*ANOVA (global effect)*	–	Friedman *χ*^2^ = 5.21	0.074
Correlation with verbal interactions	Number of evoked memories	–	Spearman rs = 0.127	0.347
	Episodicity	–	Spearman rs = − 0.016	0.906
	Expressed pleasure	–	Spearman rs = 0.29	0.029*
Negative affect	*ANOVA (global effect)*	–	Friedman *χ*^2^ = 2	0.368
Interest in activity	*ANOVA (global effect)*	–	Friedman *χ*^2^ = 1.6	0.449
TEMPau test	*ANOVA (global effect)*	–	Friedman *χ*^2^ = 5.17	0.075
Episodicity after music listening	*ANOVA (global effect)*	–	Friedman *χ*^2^ = 0.441	0.802
Episodicity – songs RW vs other songs	*Wilcoxon*	–	–	0.05
Social interactions in daily life	*Wilcoxon*	–	–	< 0.001*
	Phase 1	108 ± 18.5 (70.7–138)	–	–
	Phase 3	127 ± 12.2 (93.7–138)	–	–

*p < 0.05, statistically significant.

#### 3.1.2 Number of evoked memories through the RW

The Friedman ANOVA revealed no significant effect of the RW on the number of evoked memories (χ^2^ = 3.31, *p* = 0.191).

#### 3.1.3 Episodicity of evoked memories within the RW

There was a significant effect of the RW on the episodicity of memories score revealed by a Friedman ANOVA (χ^2^ = 6.89, *p* = 0.032). Contrast analyses indicated that episodicity of memories was significantly higher in the 9th Workshop (mean = 5.68, min = 0, max = 10, SD = 2.83) in comparison with the 5th Workshop (mean = 4.06, min = 0, max = 11, SD = 3.42) and the 1st Workshop (mean = 4.11, min = 0, max = 9, SD = 3.35) with, respectively, (*p* = 0.022 and *p* = 0.017). There was no significant difference between the 1st and the 5th Workshop.

#### 3.1.4 Expressed pleasure

The Friedman ANOVA revealed no significant effect of the RW on the score of expressed pleasure (χ^2^ = 5.21, *p* = 0.074).

#### 3.1.5 Correlation with verbal interactions

There was no significant correlation between the number of evoked memories and Verbal Interactions (rs = 0.127, *p* = 0.347).

There was no significant correlation between the episodicity of evoked memories and Verbal Interactions (rs = − 0.016, *p* = 0.906).

A significant positive correlation was found with a small effect between Expressed Pleasure and Verbal Interactions (rs = 0.29, *p* = 0.029).

#### 3.1.6 Other affects

The Friedman ANOVA revealed no significant difference in negative affect dimensions between the 1st, 5th, and 9th workshops (*p* = 0.368). The mean negative affect scores remained low throughout all sessions with means of 1.17, 1.67, and 1.17, respectively.

For interest in the activity, the Friedman ANOVA revealed no significant difference between the 1st, 5th, and 9th workshops (*p* = 0.449). The mean score for interest ratings were very high across all sessions, with means of 4.95, 4.92, and 4.89, respectively.

### 3.2 Individual interviews

#### 3.2.1 TEMPau test

We conducted a Friedman ANOVA, which revealed no significant difference in the simplified TEMPau test scores between Phase 1 (before the RW), Phase 3 (after the RW), and Phase 4 (1 month after the RW), (χ^2^ = 5.17, *p* = 0.075).

#### 3.2.2 Episodicity of evoked memories after music listening

The results of the Friedman ANOVA revealed no significant difference in the Episodicity score of evoked memories between Phase 1 (before the RW), Phase 3 (after the RW), and Phase 4 (one month after the RW), (χ^2^ = 0.441, *p* = 0.802).

#### 3.2.3 Episodicity of evoked memories, the comparison after listening to songs from the workshop and other songs

In Phase 2, after the RW sessions, we compared the episodicity of evoked memories following the listening of three songs that were most frequently used during the RW with three songs that were not used. The Wilcoxon test did not reveal a significant difference (*p* = 0.05).

#### 3.2.4 Social interactions in daily life

The Wilcoxon test revealed a significant difference (*p* < 0.001) in social interaction scores, between Phase 1, before the RW (mean = 108, min = 70.7, max = 138, SD = 18.5) and Phase 3, after the RW (mean = 127, min = 93.7, max = 138, SD = 12.2). This result indicates a statistically significant increase in social interactions following the intervention.

## 4 Discussion

The study examined whether nine musical reminiscence sessions could enhance social engagement in individuals with moderate to severe AD. Verbal interactions increased from the 5th session and remained stable through the 9th, aligning with previous findings (Platel et al., 2021). The study also explored mechanisms underlying this effect, focusing on two hypotheses: (1) memory retrieval induced by familiar music supports social interaction and (2) the pleasure generated by shared music listening acts as a driver for verbal engagement.

Our findings suggest that emotions, particularly the pleasure elicited by familiar music, represent the key mechanism facilitating social connection in individuals with AD. Indeed, the only positive correlation found with verbal interactions is associated with the level of pleasure.

We focused on interactions between participants and spontaneous participant interactions related to group discussions, reflecting genuine communication rather than just answers to the leader. It suggests that enabling AD patients to experience positive emotions and pleasure may be a core mechanism in encouraging them to interact with their social environment, regardless of their cognitive difficulties or those of their interlocutors.

Given the well-documented powerful effect of music on emotions (Juslin, 2013), even in individuals with AD (Arroyo-Anlló et al., 2019; Särkämö et al., 2012), and its capacity to generate pleasure at a neurophysiological level (Blood and Zatorre, 2001), we may associate music listening with the mood expressed by the participants.

Surprisingly, no correlation emerged between the number or episodic quality of retrieved memories and social engagement. Although we observed, in line with Platel et al. (2021), an increase in episodic quality in the 9th session, the level of detail in recollections does not seem to drive social interaction. We also did not find a significant increase in the number of memories produced, diverging from Platel et al. (2021) results. These differences may be linked to the goals and facilitation style of the sessions, as in our study, the focus was on fostering social connection while facilitating autobiographical memory retrieval. A methodological difference may also contribute: while Platel et al. (2021) analyzed entire sessions, we considered only selected excerpts (12 min per session). Longer sessions may therefore have provided more opportunities for memory production.

An interesting finding is the apparent transfer between participants’ behavior during the RW sessions and their daily lives. Specifically, the social interaction scores increased significantly after the RW. This effect may be better explained by mood improvement and the association of positive social moments with the pleasure derived from music listening (Tarr et al., 2014). Once again, memory retrieval does not appear to be the driving force behind the enhancement of social engagement, as we did not observe any significant effect of the workshops on autobiographical memory during the individual interviews conducted after the final RW session.

From a theoretical perspective, we could shed light on our results using the SEED framework proposed by Engelbrecht et al. (2021), which contains four dimensions: “Evoking emotional reactions; Eliciting physiological responses; Summoning autobiographical memories; and Defining and communicating self-identity and social relatedness”. This model describes the function of music in reminiscence therapy for older adults in general, and not specifically for those with a neurodegenerative condition. Nevertheless, we propose to analyze our results in light of this model. Indeed, Evoking emotional reactions dimension relate how music generate emotion. Across all RW sessions, we consistently observed a very low level of negative emotions and a high level of engagement in the activity. Indeed, this dimension refers to the way music generates emotions which, in this context, are generally positive, eliciting pleasure and enhancing mood (Cuddy et al., 2017). This dimension is fully consistent with our findings and appears to represent the most important pathway through which music can foster social bonding. In Engelbrecht’s model, Evoking emotional reactions is directly connected to Eliciting physiological responses via the mediation of dopamine and endogenous opioids, in line with existing neurochemical theories of music listening (Chanda and Levitin, 2013; Mas-Herrero et al., 2023; Putkinen et al., 2025). We did not assess physiological variables such as physical well-being, immune function, changes in heart rate or respiration, blood pressure, or reduced medication use. However, this physiological dimension would be of interest to investigate, as these factors play a crucial role in human health and overall quality of life.

Regarding the dimension termed Summoning autobiographical memories, which is associated with increased recall, specificity, vividness, and greater engagement with past experiences, our results did not reveal any correlation between the quantity or quality of memories and social engagement. Nonetheless, in the context of a reminiscence workshop, it is difficult to argue that memory evocation plays no role in social interaction. In fact, the observed increase in recall specificity suggests that music may function as a facilitator, enabling the retrieval of more detailed memories (Kelly and Ahessy, 2021). The absence of an increase in the number of memories produced during the RW may suggest an effect of music from the very first listening, as well as a possible ceiling effect, rather than a lack of influence.

Regarding the dimension Defining and communicating self-identity and social relatedness, our results indicate a reinforcement of social ties. Increased social interaction was observed both during the workshops and in daily life, as reported by caregivers and relatives. In one facility, a group formed during the sessions remained stable 6 months later, suggesting lasting social bonds even without explicit memory of the workshops. A particularly striking anecdote illustrates the enduring emotional impact of shared musical experiences on social connectedness: during a musical activity, one resident inquired about the RW leader 18 months after the final session. Although identity was not directly assessed, previous work has shown stability of self-identity in AD during reminiscence workshops (Platel et al., 2021).

Our study focused on the pleasure expressed during verbal interactions after music listening and at the end of the workshop. The increase in pleasure appears strongly influenced by the social context and personal relevance of the activity, both known to improve mood in people with dementia. This is consistent with the results of Cohen-Mansfield et al., (2012), who found that social, self-identity, and music-related stimuli significantly increased residents’ pleasure.

In the experiment, group sizes varied from two to four participants. Although larger groups might be expected to generate more interactions, our observations indicated that even dyads engaged frequently, suggesting that smaller group size did not limit social interactions. Notably, in one of the two-participant groups, the participants shared memories of having grown up in the same region, which appeared to further enhance engagement and interaction.

The Music Social Bond theory provides an integrative framework for understanding how music shapes social bonding and prosocial behavior through cultural and biological mechanisms (Savage et al., 2021). The predictability inherent in musical structure may facilitate the synchronization of neural activity across individuals, particularly within homologous brain regions (Kaneshiro et al., 2024; Abrams et al., 2013). Shared music listening experiences may support social bonding, presumably by fostering shared emotional uplift and synchronized actions among participants (Trehub et al., 2015; Wu et al., 2025). Our results suggest that these mechanisms may be partly preserved in individuals with AD, highlighting a promising approach to support social engagement for people living with AD.

Moreover, social reward itself may have contributed to participants ‘social engagement, as social interactions are inherently rewarding (Krach et al., 2010). These considerations highlight the importance of further investigating whether activities that enable interpersonal synchronization and promote experiences of social pleasure can foster social engagement in individuals with AD.

Taken together, these results suggest that the effects of RW on autobiographical memory and the social context provide favorable conditions for interaction, offering participants a space to share memories and connect with others. Social engagement appears to arise primarily from the pleasurable aspects of music listening, which not only elevates mood but also fosters social pleasure. This ability of music to induce positive affect may create opportunities for individuals with AD to reconnect with social enjoyment, recover shared positive emotions, and increase their motivation for interpersonal engagement. Repeated exposure to such positive social interactions and emotions may represent a process of social bond re-adaptation and an implicit encoding of these experiences.

### 4.1 Limitations

A key limitation is that personalized music was not used in the RW. Future studies should explore the impact of personalized music linked to individuals’ histories.

Additionally, the small sample size (*N* = 19) and interindividual variability (cognitive severity, autonomy, and prior musical experience) limit generalizability and may have affected group dynamics and emotional or social responses. The lack of a control group also prevents ruling out nonspecific effects (e.g., social stimulation, therapist attention, or activity novelty) as drivers of observed improvements, rather than music or reminiscence itself.

Another limitation of this study is that session timing was not consistent across all groups. Morning or afternoon sessions were scheduled to accommodate the participants’ daily routines and preferences, rather than standardized timing, which may have influenced outcomes and limits comparability between groups.

## 5 Conclusion

Repeated reminiscence workshops appear to enhance autobiographical memory retrieval during the sessions and promote social interaction both within the sessions and in participants’ daily lives. By fostering a social context where memories can be shared, these workshops create opportunities for engagement. Music listening, through its capacity to elevate mood, may play a central role by enabling individuals with AD to reconnect with social pleasure and to experience shared positive emotions. Music therapy, and more specifically musical reminiscence workshops, appears to be a valuable intervention to support social interaction in individuals living with Alzheimer’s disease. Our findings highlight the close relationship between pleasure and social engagement, suggesting that stimulating positive emotions and enjoyment within an adapted social environment constitutes a key lever to strengthen social bonds in Alzheimer’s disease.

## Data Availability

The raw data supporting the conclusions of this article will be made available by the authors, without undue reservation.

## References

[ref1] Abrams D. A. Ryali S. Chen T. Chordia P. Khouzam A. Levitin D. J. . (2013). Inter-subject synchronization of brain responses during natural music listening. Eur. J. Neurosci. 37, 1458–1469. doi: 10.1111/ejn.12173, 23578016 PMC4487043

[ref2] Arroyo-Anlló E. M. Dauphin S. Fargeau M. N. Ingrand P. Gil R. (2019). Music and emotion in Alzheimer’s disease. Alzheimer's Res. Ther. 11:1. doi: 10.1186/s13195-019-0523-y, 31391062 PMC6686394

[ref3] Ashida S. (2000). The effect of reminiscence music therapy sessions on changes in depressive symptoms in elderly persons with dementia. J. Music. Ther. 37, 170–182. doi: 10.1093/jmt/37.3.170, 10990595

[ref4] Belfi A. M. Jakubowski K. (2021). Music and autobiographical memory. Music. Sci. 4:20592043211047123. doi: 10.1177/20592043211047123

[ref5] Blood A. J. Zatorre R. J. (2001). Intensely pleasurable responses to music correlate with activity in brain regions implicated in reward and emotion. Proc. Natl. Acad. Sci. USA 98, 11818–11823. doi: 10.1073/pnas.191355898, 11573015 PMC58814

[ref6] Bourgeois C. Brigaud E. Louis E. Azzoune L. Gambonnet M. Vitou V. . (2025). Unlocking the benefits of Montessori-based reading activities in nursing home: a multiple baseline study on groups of individuals with severe dementia. Dementia (London, Engl.) 24, 436–455. doi: 10.1177/14713012241270805, 39102610

[ref7] Bradt J. Dileo C. Myers-Coffman K. Biondo J. (2021). Music interventions for improving psychological and physical outcomes in people with cancer. Cochrane Database Syst. Rev. 10. doi: 10.1002/14651858.CD006911.pub4 PMC8510511 34637527

[ref9001] Bruggencate T. T. Luijkx K. G. Sturm J. (2018). Social needs of older people: a systematic literature review. Ageing Soc. 38, 1745–1770. doi: 10.1017/S0144686X17000150

[ref8] Chanda M. L. Levitin D. J. (2013). The neurochemistry of music. Trends Cogn. Sci. 17, 179–193. doi: 10.1016/j.tics.2013.02.007, 23541122

[ref9002] Cohen J. (1988). Set correlation and contingency tables. Appl. Psychol. Meas. 12, 425–434. doi: 10.1177/014662168801200410

[ref9] Cohen-Mansfield J. Dakheel-Ali M. Marx M. S. Thein K. Regier N. G. (2015). Which unmet needs contribute to behavior problems in persons with advanced dementia? Psychiatry Res. 228, 59–64. doi: 10.1016/j.psychres.2015.03.043, 25933478 PMC4451402

[ref10] Cohen-Mansfield J. Marx M. S. Freedman L. S. Murad H. Thein K. Dakheel-Ali M. (2012). What affects pleasure in persons with advanced stage dementia? J. Psychiatr. Res. 46, 402–406. doi: 10.1016/j.jpsychires.2011.12.003, 22208995 PMC3288263

[ref11] Colverson A. J. Trifilio E. Williamson J. B. (2022). Music, mind, mood, and mingling in Alzheimer’s disease and related dementias: a scoping review. J. Alzheimer's Dis 86, 1569–1588. doi: 10.3233/JAD-215199, 35253746

[ref13] Cuddy L. L. Sikka R. Vanstone A. (2015). Preservation of musical memory and engagement in healthy aging and Alzheimer’s disease: musical memory in Alzheimer’s disease. Ann. N. Y. Acad. Sci. 1337, 223–231. doi: 10.1111/nyas.12617, 25773638

[ref12] Cuddy L. L. Sikka R. Silveira K. Bai S. Vanstone A. (2017). Music-evoked autobiographical memories (MEAMs) in Alzheimer disease: evidence for a positivity effect. Cogent Psychol. (United Kingdom) 4:1. doi: 10.1080/23311908.2016.1277578

[ref14] Del Mastro M. C. Strollo M. R. El Haj M. (2021). Music as a social bond in patients with amnesia. Behav. Brain Sci. 44. doi: 10.1017/S0140525X20000758, 34588031

[ref15] El Haj M. Allain P. (2020). Self-defining memories and their contribution to the sense of self in Alzheimer’s disease. Curr. Alzheimer Res. 17, 508–516. doi: 10.2174/1567205017666200807184942, 32851943

[ref16] El Haj M. Clément S. Fasotti L. Allain P. (2013). Effects of music on autobiographical verbal narration in Alzheimer’s disease. J. Neurolinguist. 26, 691–700. doi: 10.1016/j.jneuroling.2013.06.001

[ref17] El Haj M. Postal V. Allain P. (2012). Music enhances autobiographical memory in mild Alzheimer’s disease. Educ. Gerontol. 38:1. doi: 10.1080/03601277.2010.515897

[ref18] Engelbrecht R. Bhar S. Ciorciari J. (2021). Planting the SEED: a model to describe the functions of music in reminiscence therapy. Complement. Ther. Clin. Pract. 44:101441. doi: 10.1016/j.ctcp.2021.101441, 34247028

[ref19] Evans S. C. Garabedian C. Bray J. (2019). ‘Now he sings’. The my musical memories reminiscence programme: personalised interactive reminiscence sessions for people living with dementia. Dementia 18, 1181–1198. doi: 10.1177/1471301217710531, 28554224

[ref9006] Genguelou M. Platel H. Madiouni C. Bayard S. (2026). Assessing music engagement: adaptation, validation, and structural invariance of self-rated and informant French versions of the Music Engagement Questionnaire in middle and older aged adults. Psychol. Aesthet. Creat. Arts. doi: 10.1037/aca0000786

[ref21] Irish M. Cunningham C. J. Walsh J. B. Coakley D. Lawlor B. A. Robertson I. H. . (2006). Investigating the enhancing effect of music on autobiographical memory in mild Alzheimer’s disease. Dement. Geriatr. Cogn. Disord. 22, 108–120. doi: 10.1159/000093487, 16717466

[ref22] Jacobsen J.-H. Stelzer J. Fritz T. H. Chételat G. La Joie R. Turner R. (2015). Why musical memory can be preserved in advanced Alzheimer’s disease. Brain 138, 2438–2450. doi: 10.1093/brain/awv135 26041611

[ref23] Juslin P. N. (2013). From everyday emotions to aesthetic emotions: towards a unified theory of musical emotions. Phys Life Rev 10, 235–266. doi: 10.1016/j.plrev.2013.05.008, 23769678

[ref24] Kaneshiro B. Nguyen D. T. Norcia A. M. Dmochowski J. P. Berger J. (2024). Inter-subject correlation of electroencephalographic and behavioural responses reflects time-varying engagement with natural music. Eur. J. Neurosci. 59, 3162–3183. doi: 10.1111/ejn.16324, 38626924

[ref25] Kang H. (2012). Correlates of social engagement in nursing home residents with dementia. Asian Nurs. Res. 6, 75–81. doi: 10.1016/j.anr.2012.05.006, 25030831

[ref26] Kelly L. Ahessy B. (2021). Reminiscence-focused music therapy to promote positive mood and engagement and shared interaction for people living with dementia: an exploratory study. Voices World Forum Music Ther. 21. doi: 10.15845/voices.v21i2.3139

[ref27] Klein S. B. Cosmides L. Gangi C. E. Jackson B. Tooby J. Costabile K. A. (2009). Evolution and episodic memory: an analysis and demonstration of a social function of episodic recollection. Soc. Cogn. 27, 283–319. doi: 10.1521/soco.2009.27.2.283, 23378680 PMC3559008

[ref28] Krach S. Paulus F. M. Bodden M. Kircher T. (2010). The rewarding nature of social interactions. Front. Behav. Neurosci. 4:22. doi: 10.3389/fnbeh.2010.00022, 20577590 PMC2889690

[ref29] Lawton M.P. Van Haitsma K. Klapper J.A. (1999). Observed emotion rating scale Available online at: www.abramsoncenter.org/PRI (scale page) (Accessed March 12, 2026).

[ref9003] Leech N. Barrett K. Morgan G. A. (2004). SPSS for Intermediate Statistics : Use and Interpretation, 2nd Edn. London: Routledge. doi: 10.4324/9781410611420

[ref30] Leone E. Deudon A. Robert P. (2012). Motivation, engagement et stimulation verbale et motrice dans les démences sévères. Étude STIM-EHPAD. Rev. Neuropsychol. 4, 114–122. doi: 10.1684/nrp.2012.0216

[ref31] Mas-Herrero E. Ferreri L. Cardona G. Zatorre R. J. Pla-Juncà F. Antonijoan R. M. . (2023). The role of opioid transmission in music-induced pleasure. Ann. N. Y. Acad. Sci. 1520, 105–114. doi: 10.1111/nyas.14946, 36514207

[ref32] McKhann G. Drachman D. Folstein M. Katzman R. Price D. Stadlan E. M. (1984). Clinical diagnosis of Alzheimer’s disease: report of the NINCDS-ADRDA work group under the auspices of department of health and human services task force on Alzheimer’s disease. Neurology 34, 939–944. doi: 10.1212/wnl.34.7.939 6610841

[ref9004] Mormont E. Jamart J. Robaye L. (2012). Validity of the five-word test for the evaluation of verbal episodic memory and dementia in a memory clinic setting. J. Geriatr. Psychiatry Neurol. 25, 78–84. doi: 10.1177/0891988712445088 22689699

[ref33] Nummenmaa L. Putkinen V. Sams M. (2021). Social pleasures of music. Curr. Opin. Behav. Sci. 39, 196–202. doi: 10.1016/j.cobeha.2021.03.026

[ref9400] Ouvrard C. Berr C. Meillon C. Ribet C. Goldberg M. Zins M. . (2019). Norms for standard neuropsychological tests from the French CONSTANCES cohort. Eur. J. Neurol. 26, 786–793. doi: 10.1111/ene.13890 30575234

[ref34] Petherbridge Danielle. (2025). «Self, personhood and self-awareness: a phenomenological analysis of dementia». Phenomenol. Mind 28, 192–205. doi: 10.17454/pam-2807

[ref35] Piolino P. Desgranges B. Eustache F. (2000). La Mémoire Autobiographique: Théorie et Pratique. Marseille: Solal.

[ref36] Platel H. Eustache M.-L. Coppalle R. Viard A. Eustache F. Groussard M. . (2021). Boosting autobiographical memory and the sense of identity of Alzheimer patients through repeated reminiscence workshops? Front. Psychol. 12:636028. doi: 10.3389/fpsyg.2021.636028, 33679562 PMC7928298

[ref37] Porcelli S. van der Wee N. van der Werff S. Aghajani M. Glennon J. C. van Heukelum S. . (2019). Social brain, social dysfunction and social withdrawal. Neurosci. Biobehav. Rev. 97, 10–33. doi: 10.1016/j.neubiorev.2018.09.012 30244163

[ref38] Putkinen V. Seppälä K. Harju H. Hirvonen J. Karlsson H. K. Nummenmaa L. (2025). Pleasurable music activates cerebral Μ-opioid receptors: a combined PET-fMRI study. Eur. J. Nucl. Med. Mol. Imaging 52, 3540–3549. doi: 10.1007/s00259-025-07232-z, 40183950 PMC12316753

[ref39] Särkämö T. Laitinen S. Tervaniemi M. Nummien A. Kurki M. Rantanen P. (2012). Music, emotion, and dementia: insight from neuroscientific and clinical research. Music Med. 4, 153–162. doi: 10.1177/1943862112445323

[ref40] Savage P. E. Loui P. Tarr B. Schachner A. Glowacki L. Mithen S. . (2021). Music as a coevolved system for social bonding. Behav. Brain Sci. 44:e59. doi: 10.1017/S0140525X20000333, 32814608

[ref41] Sisco F. Taurel M. Lafont V. Bertogliati C. Baudu C. Giordana J. Y. (2000). Les troubles du comportement chez le sujet dément en institution: Évaluation à partir de l’inventaire neuropsychiatrique pour les équipes soignantes (NPI/ES). L’ Année gérontologique (Ed. française) 14, 151–171.

[ref42] Tarr B. Launay J. Dunbar R. I. M. (2014). Music and social bonding: “self-other” merging and neurohormonal mechanisms. Front. Psychol. 5:1096. doi: 10.3389/fpsyg.2014.01096 25324805 PMC4179700

[ref44] Thomas P. Lalloué F. Preux P.-M. Hazif-Thomas C. Pariel S. Inscale R. . (2006). Dementia patients caregivers quality of life: the PIXEL study. Int. J. Geriatr. Psychiatry 21, 50–56. doi: 10.1002/gps.1422, 16323256

[ref45] Trehub S. E. Becker J. Morley I. (2015). Cross-cultural perspectives on music and musicality. Philos. Trans. R. Soc. B 370:20140096. doi: 10.1098/rstb.2014.0096, 25646519 PMC4321137

[ref46] World Federation of Music Therapy. (2026). “About” Available online at: https://www.wfmt.info/about (Accessed February 27, 2026)

[ref47] Wu H. Wang D. Zhou L. (2025). Tunes that move us: the impact of music-induced emotions on prosocial decision-making. Front. Psychol. 15:1453808. doi: 10.3389/fpsyg.2024.1453808, 39850967 PMC11754231

