# Supplementary material for: Social engagement, pleasure, and memory in musical reminiscence workshops for individuals with Alzheimer’s disease
Source: Front Hum Neurosci. 2026 Apr 22;20:1803210. doi: 10.3389/fnhum.2026.1803210 (PMC13143991; doi:10.3389/fnhum.2026.1803210)
Supplement: Supplementary file 1 [file Data_Sheet_1.pdf]

Adaptation of the Observed Emotion Rating Scale (Lawton et al., 1999)

Emotion / Behavior	Never	<16 s	16–59 s	1–3 min	3–6 min
**Pleasure 1** – laughing, smiling, kissing, stroking or gently touching another person, reaching out warmly to other					
**Pleasure 2** – singing					
**Anger** – physical aggression, shouting, cursing, berating, shaking fist, drawing eyebrows together, clenching teeth, pursing lips, narrowing eyes, making distancing gesture					
**Anxiety/Fear** – screaming, repeated calling out, agitation, grimacing, repetitive agitated movements, furrowed brows, horizontal forehead lines, wringing hands, rapid breathing, wide eyes, tense facial muscles					
**General alertness** – Participating in a task; maintaining eyes contact; eyes following object or person; looking around room; responding by moving or saying something; turning body or moving toward person or object.					
