# Supplementary material for: Social engagement, pleasure, and memory in musical reminiscence workshops for individuals with Alzheimer’s disease
Source: Front Hum Neurosci. 2026 Apr 22;20:1803210. doi: 10.3389/fnhum.2026.1803210 (PMC13143991; doi:10.3389/fnhum.2026.1803210)
Supplement: Supplementary file 2 [file Data_Sheet_2.docx]

**During the individual interviews, questions were used to help stimulate recall after music listening**

This question was asked before ending the conversation about each event:” Do you have any more details to share about this event?”

Specificity clues

“Does this remind you of a particular day?”
“Did this event happen only once?”
“Do you have any more details about this particular moment that you can relive?”

Theme: School, Childhood

“Remember when you were a child in your classroom: What are you doing?”

“Who did you like to play with?”

“What games did you play during recess?”

“Did you like your teacher?”

“Did you get into mischief?”

“Did you have homework at home?”

“Was childhood an enjoyable period for you?”

“Do you remember any good childhood memories? Which ones?”

Theme: Dances, Young Adulthood

“Did you enjoy dancing?”

“Did you go to dances?”

“What kind of dance did you like?”

“Did you go with friends or a boyfriend/girlfriend?”

“Can you tell me a memory from your youth?”

“Was youth an enjoyable period for you?”

“Did anyone meet their spouse at a dance?”

“Do you have good memories from your youth? Which ones?”

Theme: Love, Older Adulthood

“Have you ever been married?”

“Did you live with a partner?”

“Did you fall in love during your adult life?”

“Did you have a love story?”

“Did you have children?”

“Did your children get married?”

“Was adulthood an enjoyable period for you?”

“Do you remember good memories from your adult life? Which ones?”
